# An unusual biconvex epidural lesion: acutely presenting extraosseous intracranial Ewing’s sarcoma

**DOI:** 10.1186/s41016-018-0139-2

**Published:** 2018-11-14

**Authors:** Kalimullah Jan, Eraj Khursheed Khan, Inamullah Khan

**Affiliations:** 1 Department of Neurosurgery, Aga Khan University Hospital, Karachi, Pakistan; 2 Present Address: Department of Medicine (Neurology), Changi General Hospital Singapore, Simei, Singapore; 3 Singapore, Singapore; 4 Department of Orthopedic Surgery, Aga Khan University Hospital, Stadium Road, Karachi, 74800 Pakistan; 5 Aga Khan University Medical College, Stadium Road, Karachi, 74800 Pakistan

**Keywords:** Brain neoplasm, Ewing’s sarcoma, Epidural, Biconvex, Extra osseous, Molecular analysis

## Abstract

**Background:**

Ewing’s sarcoma family of tumors consists of small round cell neoplasms, inclusive of primitive neuroectodermal tumor (PNET), Askin’s tumor, and PNET of the bone. Extraosseous Ewing’s sarcoma occurs commonly at bones of lower extremities and paravertebral region of the spine. It rarely presents as a primary intracranial lesion. When intracranial, it can be misdiagnosed as central PNET (e.g., medulloblastoma, pinealoblastoma, or supratentorial PNET), other intracranial lesions, or even as an epidural hematoma.

**Case presentation:**

We report the case of a 20-year-old patient who presented to the emergency department with complaints of drowsiness, headache, and fever for 1 day. On initial computed tomography (CT) scan of the brain, a right temporal biconvex epidural lesion involving squamous-temporal bone with periosteal reaction was noted.

The patient underwent urgent craniotomy, and a tumor was found and excised. Biopsy report confirmed Ewing’s sarcoma.

**Conclusion:**

Ewing’s sarcoma is a rare intracranial malignancy with only a few cases reported in literature. In a young patient with a biconvex epidural lesion, in the absence of trauma or ongoing infection, the possibility of Ewing’s sarcoma should be considered as well.

## Background

Small round cell neoplasms, including Ewing’s sarcoma, primitive neuroectodermal tumor (PNET), Askin’s tumor, PNET of the bone, and extraosseous Ewing’s sarcoma, together constitute the peripheral primitive neuroectodermal (pPNET)/Ewing’s sarcoma family of tumors [1]. Ewing sarcoma is the second most common bone tumor presenting in children. It commonly arises in the cortex of long bones and can also occur in other locations such as the ribs and vertebrae [2]. The chromosomal translocation t(11;22) (q24;q12) is pathognomonic for Ewing’s sarcoma.

Common sites of occurrence of extraosseous Ewing’s sarcoma include soft tissues and bones of the lower extremity, paravertebral, and retroperitoneal regions [1]. Extraosseous Ewing’s sarcoma rarely presents as a primary intracranial lesion. It can be misdiagnosed as central PNET, as other intracranial lesions [1], or as epidural hematoma [2]. When in the CNS, it most commonly arises as a solitary lesion from the dural surface of the brain or the spinal cord [1].

Herein, the authors report a case of a young patient with an intracranial extradural extraosseous Ewing’s sarcoma diagnosed on histopathology without t(11; 22) (q24; q12) translocation. This case is atypical, as the presentation of the case was acute and the initial neuroimaging revealed a lentiform epidural lesion, catching the neurosurgical team off-guard while performing an emergency decompressive craniotomy.

## Case presentation

A 20-year-old patient presented to the emergency department with complaints of drowsiness, headache, and fever for 1 day. There was no antecedent history of trauma or any infection focus, especially in the head and neck. On clinical examination, he was vitally stable and oriented to time, place, and person but appeared lethargic. There was a focal neurological finding of decreased muscle strength (4/5) in the left lower limb. These features were worrisome for a sinister etiology, such as raised intracranial pressure (ICP) and/or an acute central nervous system (CNS) infective process. The rest of the systemic examinations was unremarkable, and laboratory reports were within normal limits.

**Fig. 1:**
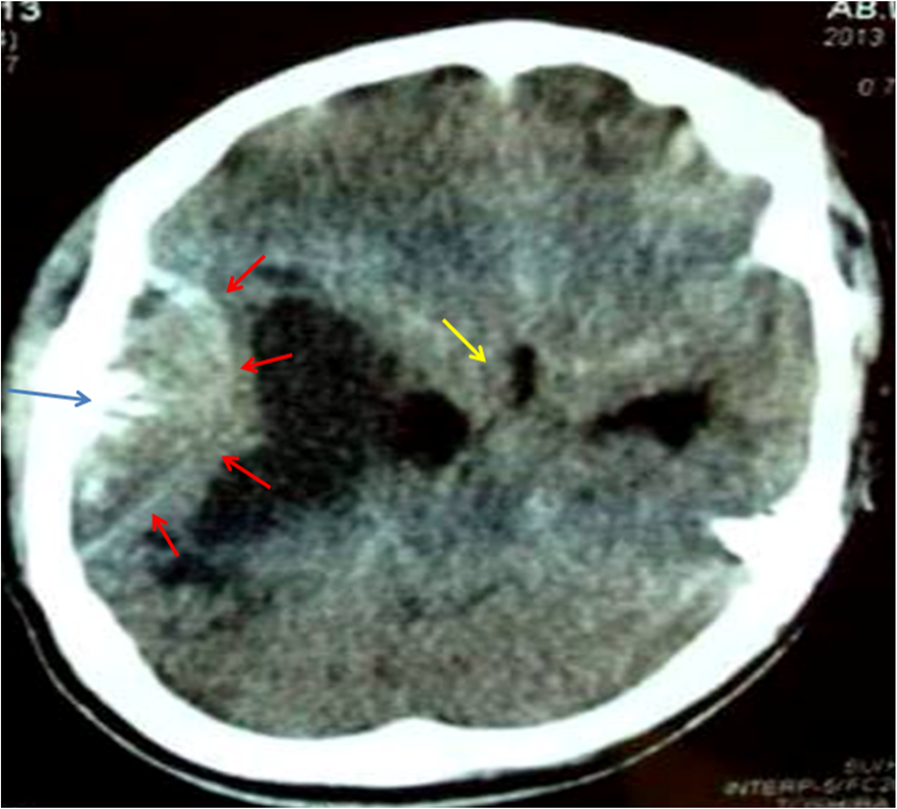
Initial non-contrasted head computed tomography (CT) scan

On initial non-contrasted head computed tomography (CT) scan, a biconvex epidural lesion was noted over the right temporal lobe, involving the squamous-temporal bone with burst-type periosteal reaction (Fig. 1). The axial non-contrast CT shows a right temporal lentiform lesion (red arrows), with burst reaction of the overlying squamous portion of the temporal bone (blue arrows). There was perilesional brain parenchymal edema with significant midline shift (yellow arrow). With the patient’s acute history, the initial differential diagnosis of this lesion included epidural empyema, epidural hematoma, oligodendroglioma, glioblastoma multiforme, or a metastatic lesion.

Given the acute presentation and obvious midline shift on the CT scan, the patient underwent an urgent decompressive craniotomy. Unexpectedly, intraoperatively, it was noted that a tumor that appeared malignant was arising from the dura mater and extending to the bone, with evidence of bone invasion. Thus, tumor excision and cranioplasty were performed.

**Fig. 2:**
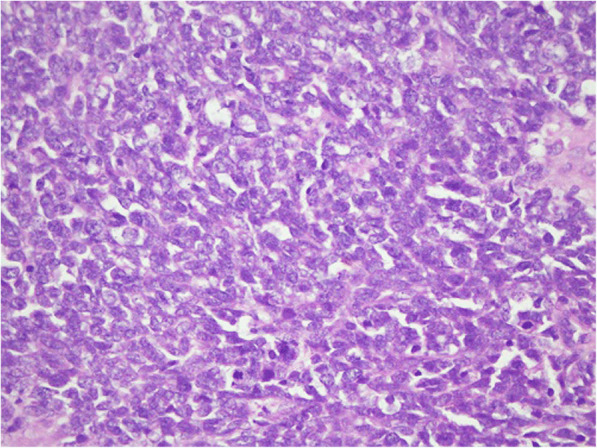
Prominent mitotic activity

Histopathology report of the biopsy sample described the presence of bone and fibro-collagenous tissue exhibiting an infiltrating neoplastic lesion arranged in sheets, nests, and rosettes. Neoplastic cells had pleomorphic hyperchromatic to vesicular nuclei, variable prominent nucleoli along with moderate to scant cytoplasm. Areas of necrosis and vascular proliferation were also seen. Prominent mitotic activity was seen as shown in Fig. 2.

Immunohistochemistry showed reactivity pattern as in Table 1.

**Table 1:** Reactivity pattern

CD99 (Mic-2)	Positive
Synaptophysin	Positive
BCL2	Positive
Desmin	Negative
EMA	Negative
CD34	Negative
CD3 (Pan-T)	Negative
CD20 (Pan-B)	Negative
CD10	Negative
TdT	Negative

A diagnosis of small round blue cell tumor was made with the morphological and immunohistochemical features confirming it as Ewing’s sarcoma/primitive neuroectodermal tumor (PNET).On fluorescent in situ hybridization (FISH), t(11;22)(q24;q12) was not detected.

Work up for metastasis, including CT scan of the chest, abdomen, pelvis, and whole spine MRI, was done and did not yield any metastatic lesions.

The patient was arranged a follow-up visit; however, he defaulted his follow-up. Three months later, he presented again with complaints of a headache and bilateral complete loss of vision and a right temporoparietal mass. He was admitted, a decompressive craniotomy and debulking of a tumor were performed, and he was referred for radio-chemotherapy.

## Discussion

Ewing’s sarcoma was first described by James Ewing in 1921 [1]. It has a male predominance and usually (70% cases) occurs in the first two decades of life [2]. Its common sites of occurrence include the bones of the lower extremity and paravertebral region of the spine [1]. A peak incidence at 15 years of age and a frequency of 1–3 per million in the western hemisphere have been noted [3].

Extraosseous Ewing’s sarcoma rarely presents as a primary intracranial lesion (CNS-EES). Typically, CNS-EES presents as a solitary lesion and is associated with the dura (70% in one review by Jantima Tanboon et al. [8]) [4, 5]. However, multifocal intracranial extradural Ewing’s sarcoma has also been described in the medical literature [4]. It can be misdiagnosed as central primitive neuroectodermal tumor (cPNET), e.g., medulloblastoma, pinealoblastoma, or supratentorial PNET, other intracranial lesions [1], or even as an epidural hematoma [2, 3].

Epidural/extradural EES, which may present as a biconvex extradural lesion, can be confused with epidural hematoma [2, 3]. This is particularly true if the lesion is detected incidentally during investigations following trauma. Thus, epidural/ extradural EES should be considered in the differential diagnosis of biconvex epidural/extradural lesions, particularly if trauma is minor and multiple lesions are detected. An unenhanced magnetic resonance imaging (MRI) of the brain may show variable isointense and hypointense signal on *T*_1_-weighted imaging while isointense and hyperintense signal on *T*_2_-weighted imaging. Post-contrast MRI scan usually reveals a heterogeneous contrast enhancement within the Ewing’s sarcoma [6].

Intracranial primary Ewing’s sarcoma is commonly associated with mild to severe headaches (the most common presenting complaint) [7]. Nausea, vomiting, diplopia, and periorbital swelling are some other symptoms that may be present.

The incidence of intracranial hemorrhage in patients with intracranial extraosseous Ewing’s sarcoma is remarkably high. Of the 17 cases reviewed by Ramon et al. [10], 7 (41%) presented with acute intraparenchymal hemorrhage [8]. However, the cause of this high rate has not been determined. There was no evidence of an intracranial bleeding in our patient.

Accurate diagnosis and differentiation between CNS-EES and cPNET is particularly important because the clinical behavior, the treatment, and the course of either tumor vary significantly [1]. In more than 97% of the cases, CNS-EES have the expression of MIC-2 gene product, CD99, on their surface which can be detected by the monoclonal antibodies O13 and HBA71 [9]. It was also positive in our case. Although positive CD99 is very indicative of Ewing’s sarcoma, definitive diagnosis is made only by the chromosomal translocation t(11, 22)(q24;q12) because CD99 can be found in other primary small cell tumors of the CNS, including ependymoma, atypical teratoid/rhabdoid tumors [4], and cPNET [1]. Chromosomal translocation t (11, 22) (q24; q12) is not found in cPNET and is pathognomonic for Ewing’s sarcoma family of tumors. Isochromosome 17q and c-MYC amplification are the common abnormalities in cPNET [1].

CNS-EES is usually treated with multimodality treatment including surgery, focal radiotherapy, and chemotherapy. Surgery is also indicated for cPNET, but radiotherapy and chemotherapy guidelines and protocols are remarkably different from those of CNS-EES [1]. For epidural EES lesions, the treatment involves surgical excision followed by adjuvant radiotherapy and combination chemotherapy with drugs such as vincristine sulfate, actinomycin-d, and cyclophosphamide (VAC) alternated with ifosfamide, cisplatin, and etoposide (ICE) [10].

The 5- and 10-year survival rates for extraosseous Ewing’s sarcoma are 69.7% and 65.2%, respectively [4]. Due to the limited number of cases, the prognosis of CNS-EES has not been fully determined; however, a more favorable prognosis has been suggested in these patients than in cPNET patients [1, 4]. Age at diagnosis, surgical treatment, micrometastasis, and circulating tumor cells are the major prognostic factors [3]. Other factors that have been suggested to result in poor prognosis are large tumor volume (> 200 ml), atypical histology, metastatic lesions, loss of p16 expression, and gains of 1q and12 [4].

## Conclusion

This case is about a young patient, who presented with acute neurological symptoms, suggesting raised ICP or CNS infection. Initial neuroimaging revealed a biconvex intracranial extradural lesion that was eventually diagnosed as extraosseous intracranial Ewing’s sarcoma.

Although, this presentation is exceedingly rare, yet in the right context, Ewing’s sarcoma can be considered as one of the differential diagnoses of Epidural brain lesion.

## Abbreviations

CNS: Central nervous system
CNS-EES: Central nervous system extraosseous Ewing’s sarcoma
CT: Computed tomography
FISH: Fluorescent in situ hybridization
ICP: Intracranial pressure
MRI: Magnetic resonance imaging
PNET: Primitive neuroectodermal tumor
